# PD-1 immunology in the kidneys: a growing relationship

**DOI:** 10.3389/fimmu.2024.1458209

**Published:** 2024-10-23

**Authors:** Ruyue Chen, Qiang Lin, Hanyun Tang, Xiaomei Dai, Lu Jiang, Ningxun Cui, Xiaozhong Li

**Affiliations:** Department of Nephrology and Immunology, Children’s Hospital of Soochow University, Suzhou, Jiangsu, China

**Keywords:** programmed death 1, programmed death ligand 1, renal cell carcinoma, glomerulonephritis, kidney transplantation, renal aging, immune-related adverse events

## Abstract

In recent years, knowledge regarding immune regulation has expanded rapidly, and major advancements have been made in immunotherapy for immune-associated disorders, particularly cancer. The programmed cell death 1 (PD-1) pathway is a cornerstone in immune regulation. It comprises PD-1 and its ligands mediating immune tolerance mechanisms and immune homeostasis. Accumulating evidence demonstrates that the PD-1 axis has a crucial immunosuppressive role in the tumor microenvironment and autoimmune diseases. PD-1 receptors and ligands on immune cells and renal parenchymal cells aid in maintaining immunological homeostasis in the kidneys. Here, we present a comprehensive review of PD-1 immunology in various kidney disorders, including renal cell carcinoma, glomerulonephritis, kidney transplantation, renal aging, and renal immune-related adverse events secondary to PD-1 immunotherapy.

## 1 Introduction

**Figure 1:**
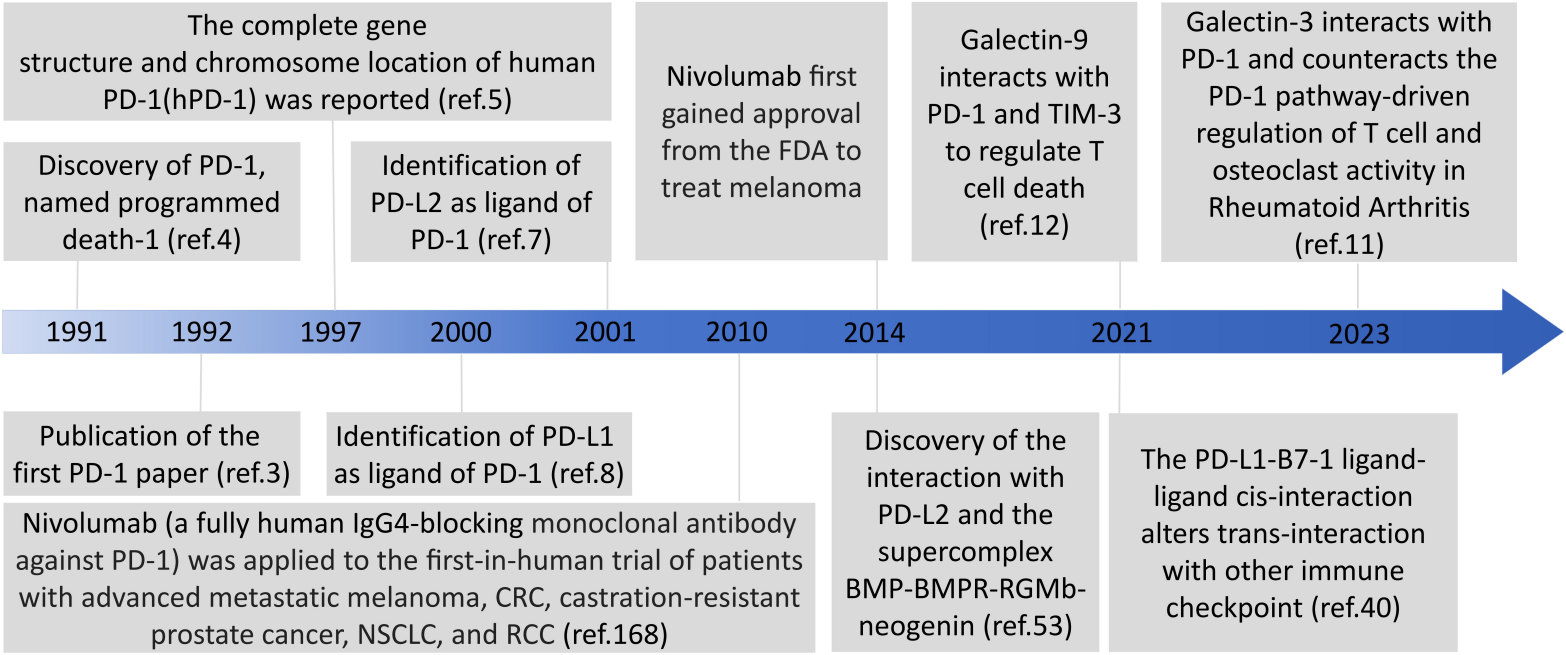
The timeline of discovery of the PD-1 receptors and ligands.

Immune checkpoint molecules are receptor-ligand pairs exerting stimulatory or inhibitory effects on immune responses. Programmed cell death 1 (PD-1) and programmed death ligands 1 and 2 (PD-L1 and PD-L2, respectively), as representative immunosuppressive checkpoints, are members of the cluster of differentiation 28 (CD28) and B7 families and constitute an inhibitory pathway, which maintains self-tolerance and affords immune homeostasis (1). PD-1 was first discovered in 1991 by Yasumasa Ishida using cDNA libraries from unstimulated and stimulated mouse T cells, and then was named programmed cell death 1 as a molecule associated with activation-induced cell death in T cells; next year, they published the first PD-1 paper in 1992 (2–4). The complete gene structure and chromosome location of human PD-1(hPD-1) was reported in 1997 (5). The identification of the interaction of PD-1 with PD-L1 in 2000 and PD-L2 in 2001 defined the PD-1 pathway (6–8). It is noteworthy that Galectin 9 (Gal-9) and galectin 3 (Gal-3) can interact with PD-1 and thus are emerging targets for cancer immunotherapy in different combinations (9–12) (**Figure 1**). The major immunosuppressive role of the PD-1 axis is responsible for the formation and maintenance of the tumor microenvironment (TME), autoimmunity, infectious immunity, transplantation immunity, allergy, and immune privilege (13, 14). Recent studies have demonstrated that PD-1 and PD-L1 overexpression on tumor cells and tumor-infiltrating lymphocytes is correlated with poor outcomes in some patients with cancer, and PD-1/PD-L1 blockade-based immunotherapy has been developed for cancers including solid tumors and hematologic malignancies (13). PD-1 was originally identified as an inducible surface receptor during programmed cell death and is mainly present on the surfaces of activated T, B, and natural killer (NK) cells, as well as dendritic cells (DCs), monocytes, macrophages, and myeloid cells (15, 16). PD-L2 expression is mainly confined to DCs, monocytes, and macrophages, whereas that of PD-L1 is more widely distributed on not only T, NK, and B cells and macrophages but also myeloid DCs, epithelial cells, vascular endothelial cells, various tumor cells, and various tumor-infiltrating cells (1). PD-L1 expression also occurs in immune-privileged sites such as the anterior chambers of the eyes, testes, and placenta (17).

**Figure 2:**
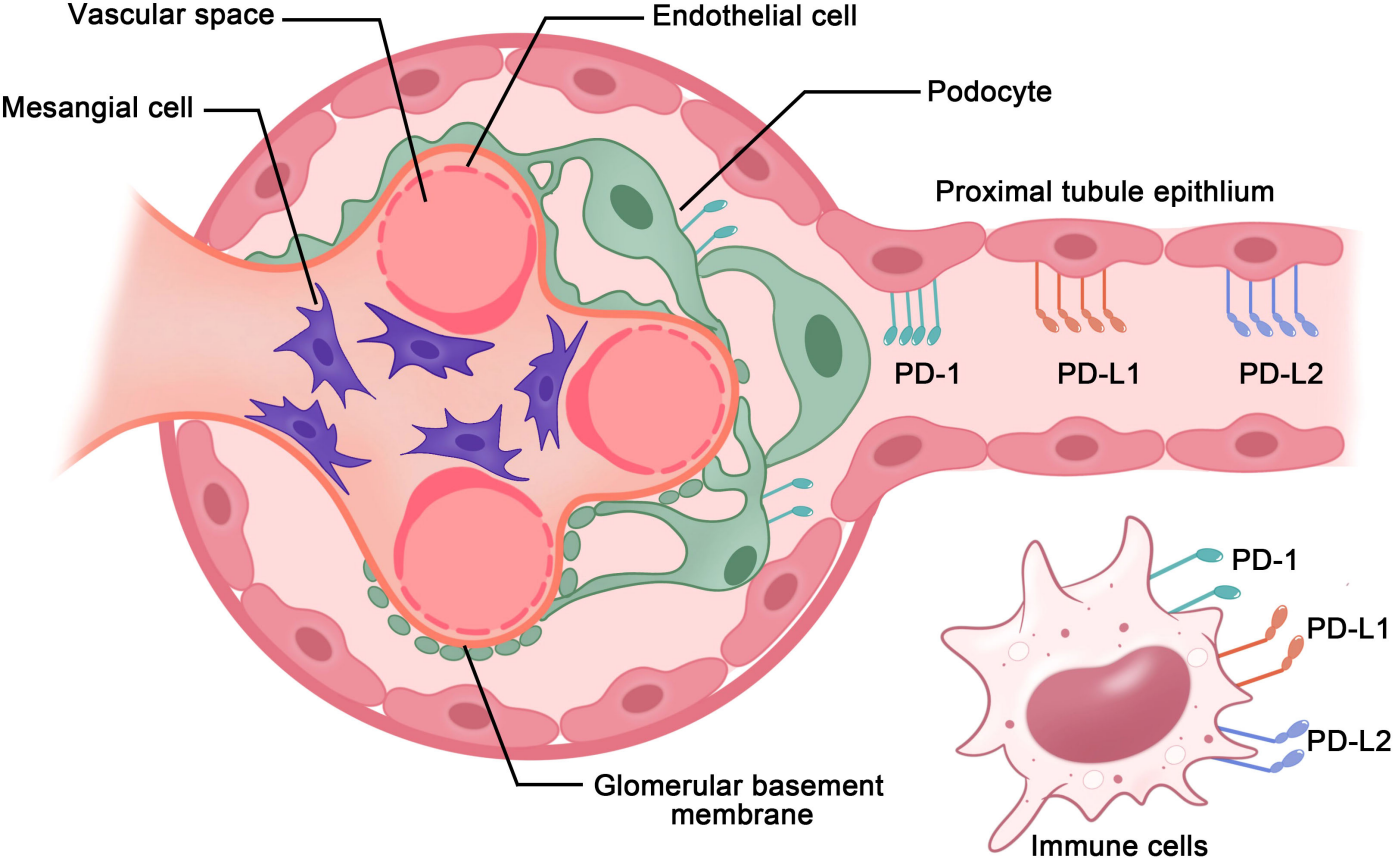
PD-1 axis in immune cells and renal parenchymal cells. PD-1 receptors and ligands on resident innate immune cells in the renal interstitium and renal parenchymal cells, including renal proximal tubule epithelial cells and podocytes, aid in maintaining immunological homeostasis within the kidneys.

**Figure 3:**
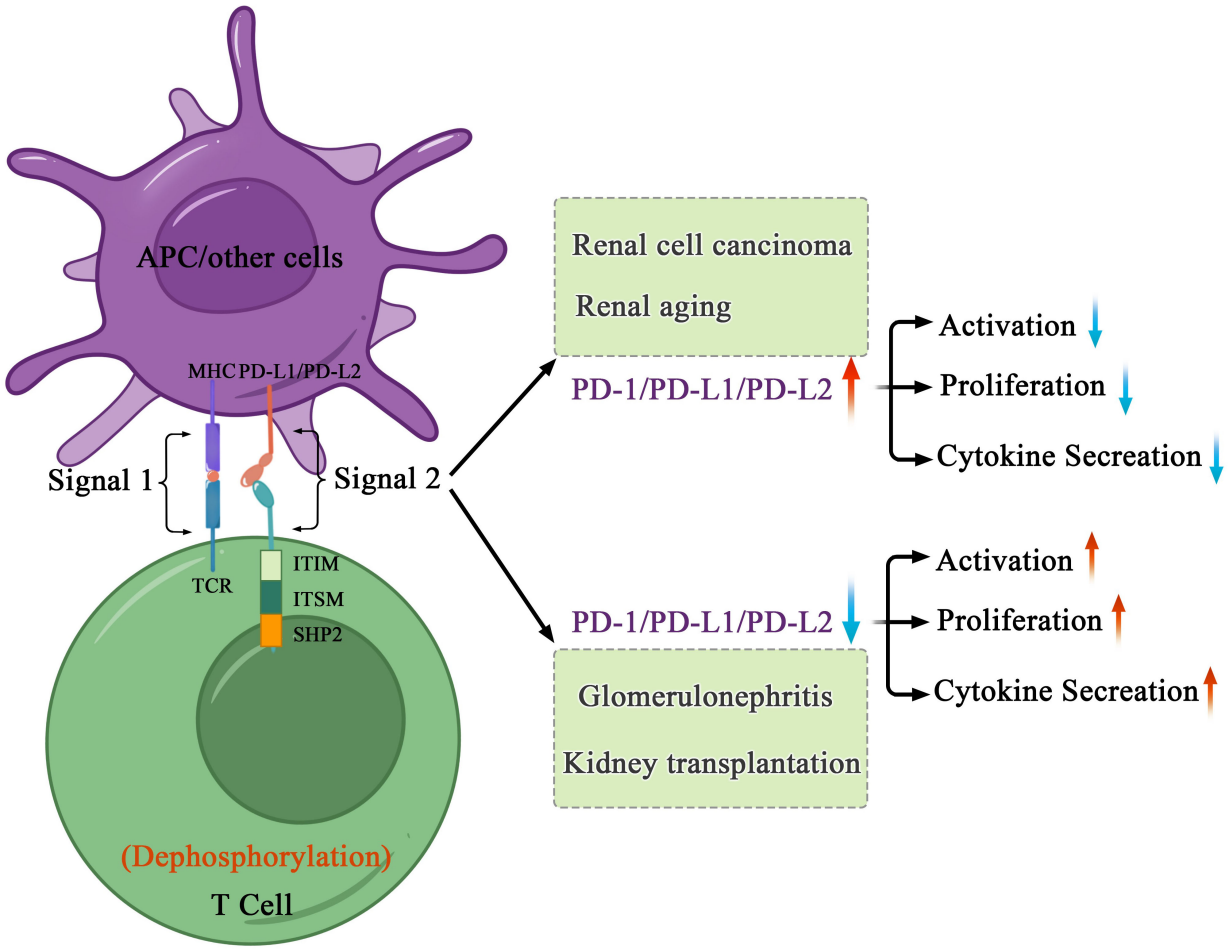
Mechanisms of PD-1-mediated inhibition in kidney health and disorders. After interacting with PD-L1 or PD-L2, PD-1 recruits the phosphatase SHP-2 in proximity to TCR, which attenuates key TCR proximal signaling. In cancers and age-related disorders, cancer or senescent cells escape the immune system because of the abnormal immune surveillance mediated by immune checkpoint molecules. In autoimmune disorders and allogenic transplantation, abnormal immune responses to self or foreign antigens expressed in transplant induce tissue damage and organ rejection.

In the kidneys, the PD-1 receptors and/or ligands have been confirmed to be present on resident innate immune cells in the renal interstitium and renal parenchymal cells, including proximal tubule epithelial cells and podocytes *in vivo* and *in vitro* (18, 19). Pippin et al. reported that in aged mouse and human kidneys, epithelial cells, including podocytes, proximal tubule epithelial cells, and tubular cells, but not glomerular mesangial and endothelial cells, demonstrated high PD-1 levels (19). Starke et al. reported that both PD-L1 and PD-L2 are expressed on human primary renal proximal tubular epithelial cells *in vivo* and *in vitro*, and PD-L1 upregulation on proximal tubular epithelial cells may attenuate acute T-cell-mediated rejection (20) (**Figure 2**). In addition, with the increasing therapeutic use of anti-PD-1/PD-L1 immune checkpoint antibodies in clinical, immune-related adverse events (irAEs) involving the kidneys are being increasingly reported (21–23). As such, PD-1 and its ligands potentially play major roles in the kidneys. Here, we provide a comprehensive review of studies on the PD-1 pathway in the kidneys, with a focus on renal cell carcinoma, glomerulonephritis, kidney transplantation, renal aging, and renal complication secondary to PD-1/PD-L1 inhibitor-related immunotherapy (**Figure 3**).

## 2 Structure and signaling of PD-1 receptor and ligands

### 2.1 PD-1

PD-1, also referred to as PDCD1 or CD279, is a type I transmembrane glycoprotein, weighing 50-55 kDa and comprising 288 amino acid residues (1). It includes an immunoglobulin superfamily domain, a 20-amino-acid stalk, a transmembrane domain, as well as an intracellular domain containing two tyrosine-based signaling motifs: immunoreceptor tyrosine-based inhibitory motif (ITIM) and an immunoreceptor tyrosine-based switch motif (ITSM) (1, 24). According to a comparative analysis of PD-1 against other proteins demonstrated, PD-1 shares 15%, 20%, and 13% similarity to the amino acid sequences of CD28, cytotoxic T lymphocyte-associated antigen-4 (CTLA-4), and inducible costimulatory molecule (ICOS), respectively (13). PD-1 belongs to the CD28 superfamily and is encoded by *PDCD1* on human chromosome 2 (1, 24). *PDCD1* comprises five exons, each serving a distinct purpose. In particular, *PDCD1* exons 1, 2, 3, and 4 encode a brief signal sequence, an immunoglobulin domain, stalk and transmembrane domains, and a truncated 12-amino-acid sequence marking cytoplasmic domain commencement, respectively; moreover, exon 5 encloses the C-terminal intracellular residues and a substantially lengthy 3′ untranslated region (UTR) (1, 25). Soluble PD-1 (sPD-1) is produced through alternative splicing of full-length PD-1 (flPD-1) transcript; only one splice variant lacking exon 3 but retaining other exons (PD-1Δx3) may encode sPD-1 (26, 27).

*PDCD1* was initially identified as a gene induced only during programmed cell death (3). PD-1 is a key immunosuppressive checkpoint, predominantly present in activated T, B, and NK cells, as well as macrophages, DCs, monocytes, and myeloid cells (1). PD-1 expression is also strong in immune-privileged regions, such as the cornea, retina, and iris-ciliary body. Its distribution is wider than that of other CD28 family members, the expression of which is restricted on T cells, which results in PD-1 demonstrating broader spectra of immune responses (17).

**Figure 4:**
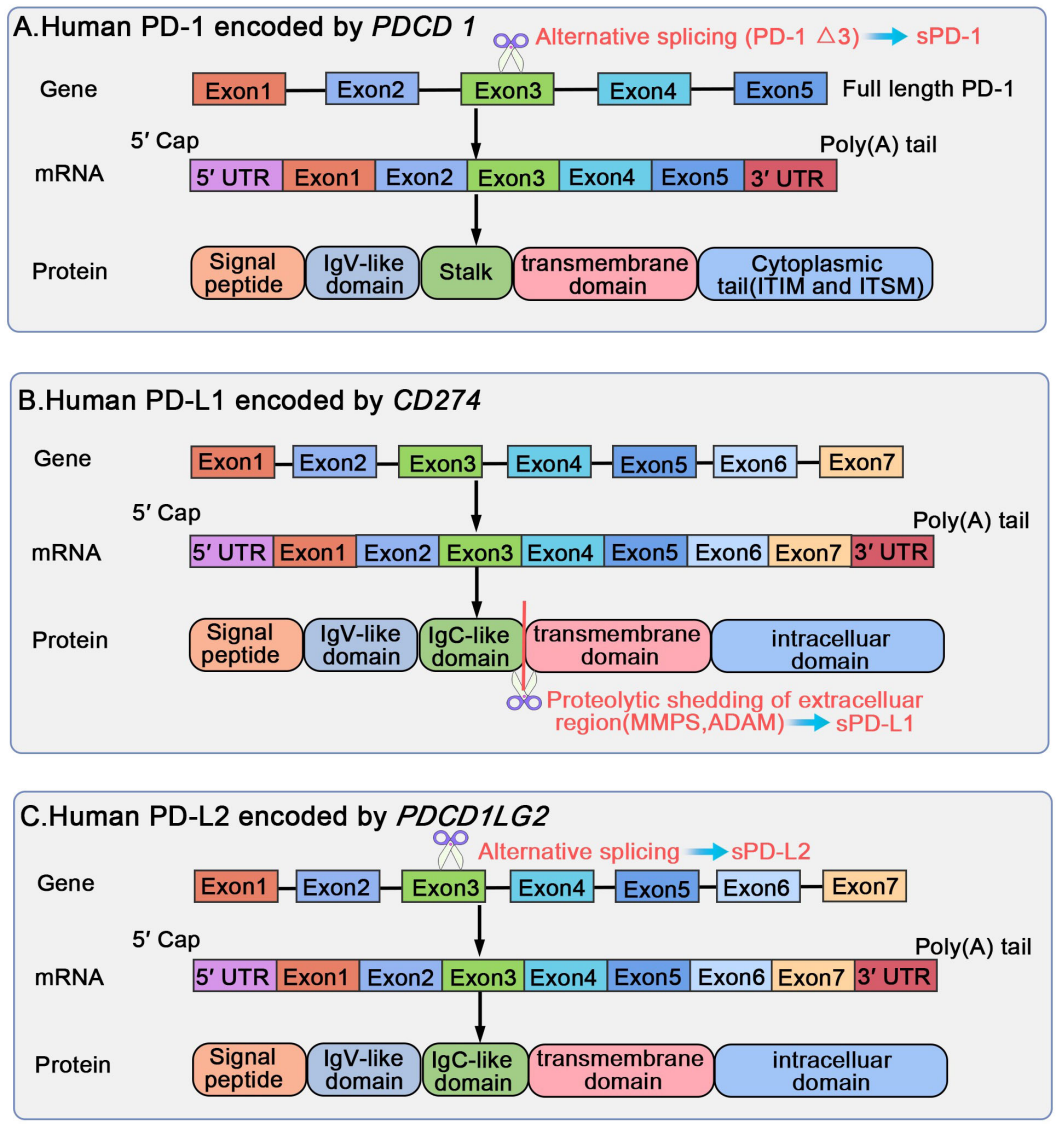
Biological regulation of human PD-1/PD-L1/PD-L2 gene, mRNA, and protein structural domains. PD-1 is encoded by *PDCD1* on human chromosome 2, including five exons, each encoding a distinct protein. sPD-1 forms through alternative slicing of the full length PD-1 transcript, and only PD-1 Δx3 may encode for sPD-1. PD-L1 and PD-L2 are encoded by *CD274* and *PDCD1LG2* located on human chromosome 9, respectively. Both *PDCD1LG2* and *CD274* present an exon organization similar to 5′ UTR, followed by a signal sequence, an IgV domain, an IgC domain, transmembrane domains, and finally cytoplasmic exons 1 and exon 2 with 3′ UTR. sPD-L1 is generated through proteolytic cleavage of mPD-L1 by various proteases, such as endogenous MMPs and ADAM. The translated protein lacks the transmembrane domain, eliminated through splicing out of exon 3; the translated protein may exist as the soluble sPD-L2.

PD-1 binds to two classical ligands: PD-L1 and PD-L2; this results in the inhibition of T-cell proliferation, activation, cytokine production, and altered metabolism, as well as cytotoxic T lymphocytes (CTLs) killer functions and eventual activated T-cell death (1). The inhibitory function of PD-1 depends on its relationship with SHP-2, a phosphatase (1). After interacting with its ligands PD-L1 or PD-L2, PD-1 becomes activated and recruits SHP-2 in proximity to T-cell antigen receptors (TCRs), which dephosphorylates protein molecules critical for TCR signaling and affects the downstream signaling pathways (28–31). The bonding of the SH2 domains of SHP-2 with ITSM in PD-1 triggers PD-1 dimerization and SHP-2 activation (1). sPD-1 functions as a PD-1 ligand blocker and suppresses the interactions of PD-1 with PD-L1 and PD-L2 and the those of PD-L1 with B7-1 (also called CD80) (27, 32). Gal-9 can interact with PD-1 on T-cell surfaces (12); this interaction plays a role in sustaining the presence of PD-1^+^TIM-3^+^ T cells and reducing Gal-9/TIM-3-induced cell death (12). Notably, studies have recently revealed the presence of direct PD-1-Gal-3 interactions, highlighting the significance of targeting Gal-3 in immunotherapy through various combination approaches (9–11) (**Figure 4**).

### 2.2 PD-L1

PD-L1, or B7 homolog 1 (B7-H1)/CD274, is a 33-kDa type I transmembrane protein, comprising 290 amino acid residues, encoded by *CD274* on human chromosome 9, and belonging to the B7 family (1, 13). *CD274* comprises seven exons (33), with exon 1 encoding a 5′-UTR, and exon 7 encoding a part of the intracellular domain and a 3′-UTR of mRNA. The first 18 amino acids form the signal peptide sequence, which is removed during protein processing (33). PD-L1 comprises a large extracellular region containing immunoglobulin (Ig) V-like and IgC-like domains, followed by a hydrophobic transmembrane domain and cytosolic tail (33). Extracellular PD-L1 can mainly be classified into free soluble (sPD-L1) and exosomal membrane-bound (mPD-L1) forms, distributed throughout the body via blood circulation (34). sPD-L1 forms through proteolytic cleavage of mPD-L1 (32). Various proteases, such as endogenous matrix metalloproteinases (MMPs) and a disintegrin and metalloproteinase (ADAM), can cleave mPD-L1, thereby enabling sPD-L1 release (32).

PD-L1 is present in various immune cells including T, B, and NK cells, as well as epithelial cells, vascular endothelial cells, antigen-presenting cells (APCs), multiple tumor cells, and tumor-infiltrating cells (1). PD-L1 overexpression can be triggered on tumor cells either by genetic alterations (innate expression) or through stimulation with interferon (IFN) γ released from effector T cells, including CD8^+^ T cells (acquired expression) (13). PD-L1 expression is also observed in immune-privileged regions such as the eye and placenta, where its overexpression begins from the fourth gestation month (17). sPD-L1 can be detected in the plasma of healthy individuals, whereas sPD-L1 levels are elevated in individuals with an autoimmune disease or cancer (26, 35, 36). In general, cancer cells and mature DCs are considered the main sources of sPD-L1 (36–38).

PD-1-PD-L1 interactions hinder T-cell receptor-mediated cytotoxicity and CD8^+^ T-cell proliferation, impeding adaptive immune response against cancer cells; this allows cancer cells to escape destruction and evade immune monitoring (39). Notably, recent studies have revealed that the PD-L1-B7-1 ligand-ligand cis-interaction alters trans interactions with other immune checkpoints; this provides newer perspectives on mechanisms underlying the currently known immune pathways and immunotherapeutic modalities (6, 40). B7-1 is a type I transmembrane protein belonging to the B7 family and existing as a monomer or homodimer. B7-1 expressed on APCs binds to CD28 and CTLA-4 on T cells, eliciting costimulatory and coinhibitory signals, respectively (41). When PD-L1 binds to B7-1 in a cis configuration, PD-L1 cannot engage PD-1 (6, 40). The PD-L1-B7-1 cis-interaction disrupts the B7-1 homodimer and reduces its avidity to CTLA-4, thereby reducing B7-1 transendocytosis (6, 42). The binding of B7-1 to PD-L1 does not prevent the interaction of B7-1 with CD28, consequently leading to the formation of a trimeric complex (6). However, the effects of the PD-L1-B7-1 cis-heterodimer on the B7-1-CD28 interaction remain inconclusive due to conflicting reports (40). Sugiura et al. demonstrated that elimination of PD-1 restriction via targeting the cis-PD-L1-B7-1 duplex effectively alleviated autoimmune disease symptoms in murine models with arthritis, multiple sclerosis, or Sjögren’s syndrome (43). Moreover, the relative levels of PD-L1 and B7-1 affected the overall outcome (6). sPD-L1 retains binding ability and inhibitory properties identical to those of mPD-L1 and can bind with PD-1 or B7-1 (26, 32) (**Figure 4**).

### 2.3 PD-L2

PD-L2, also referred to as B7-DC or CD273, is a type I transmembrane protein, belonging to the B7 ligand family, comprising 270 amino acid residues, and encoded by *PDCD1LG2* located on human chromosome 9. This gene is oriented in a direction identical to that of *CD274*, which encodes PD-L1, 42 kb apart (44–46). Both *PDCD1LG2* and *CD274* present similar exon organization with a 5′-UTR, followed by a signal sequence, an IgV domain, an IgC domain, transmembrane domains, and cytoplasmic exons 1 and 2 with 3′-UTR (46). Two novel variants of human PD-L2, other than the full-length isoform encoding the common PD-L2 mRNA (type I), have been discovered in activated leukocytes (1): A type II splice variant forms through the exclusion of exon 3 via splicing and retention of all other segments without frame alteration; the resulting protein lacks the IgC-like domain but retains the Ig constant-like domain, making it shorter in the extracellular area (2). A type III variant forms through the splicing of exon 3 into an alternative acceptor site located 5 bp downstream of the conventional site, which causes a frameshift; the translated protein, lacking a transmembrane domain, might exist in a soluble form as soluble PD-L2 (sPD-L2) (27, 47).

PD-L2 is mainly present on APCs, such as macrophages and DCs, and its expression can be induced in other immune and nonimmune cells by various microenvironmental stimuli, particularly T helper 2-related cytokines (48). In contrast, both immune and nonimmune cells express PD-L1 (45). PD-L2 expression has been detected in patients with various malignancies, and it is considered a predictor of a worse prognosis (45, 48, 49). In human tumor samples, PD-L2 and PD-L1 expression is typically correlated; however, in some subsets of patient samples, PD-L2 expression is also present in the absence of PD-L1 expression (50).

Compared with that on PD-1 and PD-L1, research on PD-L2 as a therapeutic target and predictive biomarker has been scant. Despite sharing the same receptor PD-1 and having 37% sequence homology with PD-L1, PD-L2 and PD-L1 exhibit variations in affinity and tissue expression (45). Studies have indicated that PD-L2 demonstrates a binding affinity to PD-1 that is twofold to sixfold higher than PD-L1 (44, 48, 49). Moreover, the role of PD-L2 has been highlighted in allergy and tolerance studies. Another crucial binding partner of PD­L2 is the recently discovered repulsive guidance molecule b (RGMb) (6, 51, 52). RGMb, a glycosylphosphatidylinositol-anchored protein, belongs to the repulsive guidance molecule family alongside repulsive guidance molecules a and c (6). Notably, RGMb acts as a coreceptor for bone morphogenetic protein 2 (BMP2) and bone morphogenetic protein 4 (BMP4), as well as neogenin, forming the supercomplex BMP-BMPR-RGMb-neogenin within the same cell membrane. PD-L2 can interact with this supercomplex in a trans configuration to regulate the downstream pathways (6, 51, 53). However, further relevant research on fully understanding the functional role of PD-L2 within this supercomplex is warranted (**Figure 4**).

### 2.4 Gal-9

Gal-9 is a β-galactoside-binding lectin encoded by *LGALS9* located on human chromosome 17, long arm at locus 11.2 (17q11.2) in humans (54). It is a 34-39-kDa tandem repeat galectin, consisting of two carbohydrate-recognition domains (CRDs), N- and C-terminal CRDs, with similar but distinct specificities for glycans joined with a linker domain (54–56). Full-length Gal-9 is 355 amino acids long, and *LGALS9*, consists of 11 exons, and forms many splice variants undergoing posttranscriptional splicing, such as Gal-9Δ5, Gal-9Δ5/6, Gal-9Δ5/10, and Gal-9Δ5/6/10 (54). Gal-9 has been discovered in many tissues; it was first discovered and named in mouse embryonic kidneys (57). T cells can stimulate Gal-9 release in different human cancer cell lines originating from solid malignant tumors through two distinct pathways. The first pathway involves translocation of Gal-9 onto the cell surface, followed by its proteolytic shedding, whereas the second pathway depends on autophagy, followed by lysosomal secretion; because Gal-9 lacks a secretion sequence, both these pathways require a protein carrier or trafficker (58). Gal-9 belongs to the lectin family and thus functions via receptors such as T-cell immunoglobin and mucin domain-containing protein 3 (TIM-3), V-domain Ig suppressor of T-cell activation (VISTA), and PD-1 in CTLs (9, 12, 58, 59).

TIM-3 is a glycoprotein expressed on different immune cells such as T and B cells, macrophages, monocytes, DCs, NK cells, mast cells, and APCs (60–63). TIM-3 is involved in immune response and tolerance regulation. Interactions between Gal-9 and TIM-3 can lead to immunostimulatory or immunoinhibitory effects, depending on the type of immune cells involved (60). On T cells, a TIM-3-Gal-9 interaction leads to the weakening of the T helper 1-mediated immunity and T-cell apoptosis, resulting in an inhibition of the immune system. In contrast, on NK cells and DCs, this interaction leads to an immunostimulatory effect (60, 61). In addition, TIM-3 ligands also include Psdter, high mobility group box 1, and carcinoembryonic antigen-associated cell adhesion molecule 1, which have different effects after binding to different ligands on immune cells (64).

VISTA, also known as PD-1 homolog or B7-H5, belongs to the B7 family. Structural analysis has demonstrated that the IgV domain of VISTA has sequence homology with both CD28 and the B7 family proteins, whereas the full-length VISTA demonstrates the highest identity with PD-1 (20, 65). Yasinska et al. reported that VISTA interacts with Gal-9 with relatively strong affinity, without preventing interactions between Gal-9 and TIM-3 (59). Soluble VISTA released by acute myeloid leukemia cells enhances the effects of Gal-9, most likely by forming multiprotein complexes on the T-cell surfaces and possibly creating a molecular barrier Gal-9, which results in changes in the plasma membrane potential of T cells; this leads to activation of granzyme B inside CTLs, followed by their apoptosis (59). Furthermore, human VISTA has two confirmed binding partners with immunosuppressive functions, P-selectin glycoprotein ligand 1 and V-set and Ig domain-containing 3, and one less-confirmed partner, V-set and Ig domain-containing 8 (66). VISTA activity imposes quiescence in mammalian myeloid cells and naïve T cells and inhibits T-cell activation and cytokine production; this suggests that VISTA is a promising target for combination cancer immunotherapy (66).

Recent research has indicated that Gal-9 is a PD-1-binding protein (9, 12). The binding of Gal-9 to PD-1 is highly specific and is primarily mediated by the CRD of Gal-9 and the N116-linked glycan of PD-1. Nevertheless, this association does not affect the binding of PD-1 to PD-L1 (its natural ligand) or pembrolizumab and nivolumab (the therapeutic antibodies) (12). The interactions of PD-1 with Gal-9 and TIM-3 can attenuate Gal-9/TIM-3-induced apoptosis of PD-1^+^TIM-3^+^ T cells in cancers; this result provides a newer insight into the intricate conflict between cancer cells and the immune system (12). Moreover, similar to PD-1 and its ligands (PD-L1 and PD-L2), Gal-9 expression and secretion are upregulated by IFN signaling (67).

### 2.5 Gal-3

Gal-3, a lectin with a preference for β-galactoside-containing carbohydrates, is a structurally unique galectin family member (68). Full-length Gal-3, consisting of 250 amino acids, is encoded by *LGALS3* on human chromosome 14 (68). Galectins can be classified into three types based on the number and arrangement of CRDs: prototype (a noncovalently bound isoform dimer), tandem repeat type (a covalently attached heterodimer), and chimeric type (consisting of a C-terminal CRD and an N-terminal peptide chain) (69, 70). Gal-3 is the only chimeric protein with a C-terminus CRD linked to an N-terminal domain rich in proline, glycine, and tyrosine, which can be oligomerized by the N-terminal CRD according to environmental conditions (69, 71). The N-terminal domain targets specific cellular targets, the repetitive collagen-like sequence serves as a substrate for MMP, and the C-terminal domain contains the carbohydrate-binding region (72, 73). The N-terminal peptide harbors two crucial phosphorylation sites: Ser6 (the major site with 90% phosphorylation) and Ser12 (the minor site with 10% phosphorylation) (68, 74). Both these sites are vital in facilitating nuclear export of Gal-3, and they are indispensable for the antiapoptotic functions of cytoplasmic Gal-3 (68, 75, 76). Thus far, the binding partners of Gal-3 have been reported to include lymphocyte-activation gene 3 (LAG-3), CD45, a galectin-3-binding-protein (Gal3BP), and PD-1 (9).

LAG-3 is an inhibitory receptor, highly expressed by exhausted T cells, as well as a promising immunotherapeutic target. Thus far, >20 LAG-3-targeting therapeutics are under clinical trials, and a fixed-dose combination of anti-LAG-3 and anti-PD-1 has been approved for unresectable or metastatic melanoma treatment (77). The canonical ligand of LAG-3 is a major histocompatibility complex class II protein (77, 78). Additional ligands for LAG-3 include Gal-3, liver and lymph node sinusoidal endothelial cell C-type lectin, fibrinogen-like protein 1, α-synuclein preformed fibrils, and the TCR-CD3 complex (77). In a pancreatic ductal adenocarcinoma model, Gal-3 was noted to mediate suppression of effector CD8^+^ T-cell function by binding to LAG-3 (9, 79). Moreover, a blockade of the Gal-3/LAG-3 axis has been validated in T cells in patients with multiple myeloma (9, 80).

CD45 is a transmembrane protein tyrosine phosphatase receptor type C, which is expressed exclusively in leukocytes; it has opposing effects on T-cell receptor activity (81, 82). Extracellular Gal-3 induces T-cell apoptosis by binding to CD45, whereas intracellular Gal-3 inhibits the apoptotic process by binding to BCL-2 (83, 84). Gal3BP, also known as tumor-associated antigen 90K or Mac-2-binding protein, is a multifunctional secreted glycoprotein encoded by *LGAL3SBP* involved in cell-cell and cell-matrix interactions, upregulated in patients with cancer or a viral infection, including HIV-1, HCV, or SARS-CoV-2 infection, with a key role in regulating the antiviral immune response (85). Interactions between Gal3BP and Gal-3 can trigger IL-6 expression and release in various cells, such as bone marrow stromal cells, neuroblastoma cells, and macrophages; this is because Gal3BP downregulation leads to decreased IL-6 expression, and Gal3BP/Gal-3-mediated induction of IL-6 involves the Ras-Mek-Erk1/2 pathway (85–88). In a triple-negative breast cancer model, tumor-secreted Gal-3 and a Gal-3-binding protein have been noted to form a complex that interacts with the CD45 receptor on T cells and induce expansion of regulatory T cells (9, 83).

Notably, PD-1/PD-L1 have recently been demonstrated to directly interact with Gal-3, highlighting the importance of Gal-3 as an emerging immunotherapy target in different combination modalities (9–11). Pedersen et al. reported that PD-1 and Gal-3 can be present on mononuclear cells in blood and synovial fluid and that Gal-3 can inhibit PD-1 signaling when PD-L1 is present (11). Clinical trials on the use of Gal-3 inhibitors to improve the effectiveness of anti-PD-1 therapy for metastatic melanoma and head and neck squamous cell carcinoma are underway (11, 89). Much of knowledge of these immunosuppressive molecules has come from murine studies and lot needs to be worked out in human diseases.

## 3 PD-1 pathway and kidney diseases

### 3.1 Renal cell carcinoma

Renal cell carcinoma (RCC), the most common form of kidney cancer, is associated with poor prognosis, with 25%-30% of the patients being diagnosed at the metastatic stage, and approximately 40% of the patients demonstrating recurrence after surgical excision (90, 91). Therefore, improved treatment modalities that may reduce the risk of recurrence in patients with advanced-stage disease, mainly including antiangiogenics and targeted immunotherapy, are under investigation. RCC presents as a heterogenous tumor, and it can be classified into several subtypes with unique characteristics. Clear-cell RCC (ccRCC) is the predominant subtype, accounting for approximately 75%-83% of all RCC cases (90, 92). Several other renal epithelial malignancies are collectively referred to as non-ccRCC; they include papillary RCC (pRCC), chromophobe RCC (chRCC), translocation RCC, collecting duct carcinoma, and unclassified RCC (93). RCC is considered immunogenic, characterized by the presence of abundant tumor-infiltrating immune cells, including CD8^+^ and CD4^+^ T cells, NK cells, and myeloid cells with the characteristics of macrophages and neutrophils (94). These tumor-infiltrating cells block the development of antitumor immune response in the TME, including inhibition of the activity of effector T cells and APCs via the upregulation of suppressive factors such as checkpoint molecules (93). Immune infiltration of tumors is closely associated with clinical outcomes in RCC. An increase in the proportion of regulatory T cells and the levels of the immunomodulator molecules CTLA-4 and LAG-3 worsens outcomes in ccRCC. Moreover, M2 macrophages and PD-L2 are associated with a poor prognosis in pRCC (94).

The utility of immune checkpoints as predictive biomarkers has been investigated extensively. PD-1 and PD-L1 are associated with worse clinical outcomes in RCC (92). An increase in PD-1 expression on CD14^bright^ myelomonocytic cells, effector T cells, and NK cells is significantly correlated with the disease stage in patients with RCC (95). In addition, a rapid reduction in PD-1 expression on these cells can occur within weeks after surgical tumor resection (95). Measuring PD-1 levels in peripheral blood may be a useful indicator of disease progression and response to anti-PD-1. Among RCC tumor specimens, approximately 10%-57% have been observed to be positive for PD-L1 and associated with worse clinical outcomes (96–98). Abbas et al. retrospectively analyzed intratumoral expression of PD-L1 in patients with ccRCC and reported a significant association of PD-L1 positivity with poor clinical prognosis parameters and decreased overall survival (97). Moreover, PD-L1 expression does not differ between the various histologic subtypes of RCC (98). PD-L2 expression in RCC has also been found to be associated with adverse clinicopathological features. Shin et al. detected PD-L2 in 49.6% of the samples, with the highest frequency noted for pRCC; the positivity was significantly correlated with short progression-free and cancer-specific survivals in patients with ccRCC (98). Erlmeier et al. reported high PD-L2 expression in 28.4% of their chRCC cases and a significant difference in overall survival dependent on PD-L2 expression (99).

The targeted immunotherapeutic modalities have rapidly developed for RCC in recent years. The application of various immune checkpoint inhibitors (ICIs), alone or in combination, has demonstrated strong responses in some patients with RCC. In the phase III CheckMate 025 trial, a 23% objective response rate was noted in advanced RCC patients treated with the anti-PD-1 nivolumab; in these patients, nivolumab demonstrated efficacy, safety, and tolerability superior to those of everolimus (100). In the phase II Keynote-427 study, single-agent pembrolizumab monotherapy (a humanized monoclonal anti-PD1 antibody) resulted in an overall objective response of 36% in patients with advanced ccRCC, and 68.2% of the patients demonstrated a decrease in the number of target lesions (101). Three recent phase III trials, namely Checkmate 214, Keynote-426, and Javelin Renal 101, have provided three novel, U.S. Food and Drug Administration-approved regimens for treating ccRCC: nivolumab + ipilimumab, pembrolizumab + axitinib, and avelumab + axitinib, respectively (102). However, some patients with RCC may not benefit from checkpoint blockade. Trials assessing appropriate treatment regimens for enhancing antitumor immune responses are underway.

### 3.2 Glomerulonephritis

PD-1 and its ligands, PD-L1 and PD-L2, elicit inhibitory signals to terminate or attenuate the immune response and thus play a significant role in autoimmunity (17). Systemic lupus erythematosus (SLE) is a systemic autoimmune disorder, presenting as immune tolerance loss and immune cell hyperactivity. Lupus nephritis, a common, severe manifestation of SLE, is characterized by subendothelial immune complex depositions, subepithelial immune complex depositions, or both in the afflicted kidney, resulting in extensive injury and nephron loss (103). Dysregulated cell signals in SLE may identify pathways involved in controlling the PD-1 response (104). Expression of PD-1, PD-L1 and PD-L2 has been determined in tissue, cell and serum expression in SLE patients (105). George K. Bertsias et al. reported that PD-1 staining was detected in the glomeruli in 8 of 13 samples from patients with lupus nephritis compared with 0 of 9 control samples; similarly, PD-1 was detected in renal tubules in 6 of 13 samples from patients with lupus nephritis but in 0 of 9 control samples; all 8 PD-1+ lupus nephritis samples were also stained positive for CD3 expressed in the glomeruli and tubulointerstitial region, suggesting a correlation between PD-1 and CD3+ T cell infiltrates in lupus nephritis (106). Compared with healthy controls, patients with SLE demonstrate higher PD-1^+^ and lower PD-L1^+^ immune cell percentages among peripheral blood mononuclear cells (107). The inhibitory PD-1/PD-L1/2 pathway can also be restricted by the soluble form of PD-1 (sPD-1). SLE patients with high disease activity demonstrate significantly higher serum sPD-1 levels than those with low disease activity; this decrease occurs in accordance with disease amelioration after treatment (108). In a murine SLE model, the application of anti-PD-L1 could alleviate proteinuria and prolong survival via the suppression of CD4^+^ T-cell activation, T helper 17 differentiation, autoantibody-containing immune complex deposition in the kidneys, and cytokine production (including that of IFN-γ, IL-17, and IL-10) (109). In addition, blood sPD-L2 levels were lower in patients with SLE than in healthy individuals, and they are positively correlated with complements 3 and 4 (110). Guiteras et al. designed a human fusion recombinant protein (Hybri) with two domains: CTLA-4 (to block the CD28-CD80 costimulatory pathway) and PD-L2 (to exacerbate the PD-1-PD-L2 coinhibitory pathway); this protein prevented the progression of proteinuria and anti-dsDNA to levels similar to those of cyclophosphamide, as well as reduced the histological score, infiltration of B and T cells and macrophages, and immune deposition, in NZB/WF1 and MRL/lpr mouse models of lupus nephritis (**Table 1**) (111). Besides, the immune checkpoint blockade targeting PD-1 inhibitory pathways has been a successful treatment in several cancers; the blocking of these inhibitory immune checkpoint receptors is also associated with further irAEs that can resemble lupus-like autoimmune diseases (112, 113). Therefore, PD-1 and its ligands have been identified as immune regulatory molecules implicated in SLE pathogenesis and development (104).

**Table 1:** Interventions targeting the PD-1/PD-L1 pathway for treatment of noncancer disorders.

Disease	Targeted therapy	Target	Species	References
SLE	PD-L1-Ig	PD-1	Mice	(109)
SLE	Hybri	CTLA-4 and PD-L2	Mice	(111)
Arthritis	Anti-CD80 antibodies (TKMG48)	cis-PD-L1-CD80 duplex	Mice	(43)
Multiple sclerosis	Anti-CD80 antibodies (TKMG48)	cis-PD-L1-CD80 duplex	Mice	(43)
Sjögren’s syndrome	Anti-CD80 antibodies (TKMG48)	cis-PD-L1-CD80 duplex	Mice	(43)
Colitis	Anti-PD-1 agonist mAbs (HM266)	PD-1	Mice	(130)
aGVHD	Anti-PD-1 agonist mAbs (HM266)	PD-1	Mice	(130)
FSGS	Anti-PD-1 antibody	PD-1	Mice	(19)
Aged-kidney	Anti-PD-1 antibody	PD-1	Mice	(19)
Aged-liver	Anti-PD-1 antibody	PD-1	Mice	(134)
Pulmonary fibrosis	Anti-PD-L1 mAb	PD-L1	Mice	(144)
CAEBV	Sintilimab	PD-1	Human	(163)
CAEBV	Pembrolizumab	PD-1	Human	(164)
CAEBV	Sintilimab	PD-1	Human	(164)
CAEBV	Nivolumab	PD-1	Human	(164)
CAEBV	Sintilimab	PD-1	Human	(165)
CAEBV	Sintilimab	PD-1	Human	(166)
EBV-HLH	Sintilimab	PD-1	Human	(166)
EBV-HLH	Nivolumab	PD-1	Human	(167)

SLE, systemic lupus erythematosus; aGVHD, acute graft versus host disease; FSGS, focal segmental glomerulosclerosis; PD-L1, programmed death ligand 1; CD80, cluster of differentiation 80; PD-1, programmed death 1; CTLA-4, cytotoxic T lymphocyte-associated antigen 4; PD-L2, programmed death ligand 2.

Autoimmune glomerulonephritis occurs as a consequence of autoantibody and T-cell effector functions, which target either antigens intrinsic to the glomeruli or nonspecific antibodies that become trapped and accumulate in the glomeruli (15). Grywalska et al. reported that the frequencies of PD-1- and PD-L1-positive T and B cells were higher in patients with proliferative glomerulonephritis (PGN) (seven with IgA nephropathy, and three with membranoproliferative glomerulonephritis) than in patients with non-PGN or in control individuals (four with minimal change disease, and six with membranous glomerulonephritis) (114). Studies on experimental autoimmune glomerulonephritis have demonstrated that stimulation of PD-1 using PD-L1/Fc fusion protein leads to a significant reduction in albuminuria, serum urea, serum creatinine, crescent formation, and tubular damage, as well as the numbers of glomerular macrophages, CD4^+^ and CD8^+^ T cells, and PD-1^+^ cells (115). However, studies on acute experimental foreign antigen-induced circulating immune complex glomerulonephritis have shown that the endogenous PD-1/PD-L pathway elicited by antimouse PD-1/PD-L1/PD-L2 antibody administration does not result in any significant pathological changes (116). IgA nephropathy (IgAN), the most common form of primary glomerulonephritis worldwide, is characterized by increased amounts of aberrantly glycosylated IgA1 (Gd-IgA1) present in patient serum and glomerular immune deposits (117). T cells promote IgA production and mediate the course of IgAN (118). In a study, the percentages of different subsets of circulating PD-1^hi^CXCR5^−^ T and CD138^+^ B cells were significantly higher in patients with IgAN than in healthy individuals, and the percentage of these cells was correlated with disease severity (119). Henoch-Schönlein purpura (HSP), also an IgA-mediated systemic small-vessel vasculitis, was noted to result in renal manifestations in 40%-50% of the patients (120). Moreover, the number of circulating CD4^+^CXCR5^+^PD-1^+^ T follicular helper cells was significantly higher in patients with HSP nephritis than in healthy individuals, and it was negatively correlated with 24-hour urinary protein levels (121).

### 3.3 Kidney transplantation

Allogenic transplantation is associated with allograft rejection risk due to exposure to foreign antigens, and a major factor triggering organ rejection is T-cell activation during immune allorecognition (122). PD-1 and its ligands are crucial regulators of T-cell activation and self-tolerance mechanisms. Both experimental and clinical evidence has indicated that PD-1 signaling can modulate transplant rejection. Murine models of kidney transplantation demonstrated that >90% of CD8^+^ T cells and approximately 75% of CD4^+^ T cells in early infiltrates of renal transplants express PD-1, and blocking PD-1-PD-L1 interactions using anti-PD-L1 early after transplantation can increase the amounts of T-cell infiltrates, resulting in terminal acute rejection (123). Besides, Zihuan Luo et al. developed a membrane-anchored-protein PD-L1 (map-PD-L1), which effectively anchored onto the surface of rat glomerular endothelial cells and could bind to PD-1 promoting T cell apoptosis and inhibiting T cell activation; ex vivo perfusion of donor kidneys with map-PD-L1 significantly protected grafts against acute injury without using any immunosuppressant in kidney transplantation models (124). Studies in humans have indicated that PD-L1, PD-L2, and PD-1 mRNA and protein expression is upregulated in biopsies of patients with renal allograft rejection compared with that in their pre-transplant biopsies, and a blockade of PD-L1 on tubular epithelial cells results in a significant increase in the proliferation of CD4^+^ T cells and cytokine production in CD4^+^ and CD8^+^ T cells *in vitro* (20). Melendreras et al. observed that high levels of soluble co-signaling molecules (sCD30, sCD40, sCD137, sCD40L, sPD-1, and sPD-L1) in the sera of kidney transplant recipients determined were strongly associated with a higher risk of graft failure at 3 months after transplantation than at 6 years after transplantation, suggesting that these soluble molecules may be useful biomarkers for predicting long-term graft function (125). Collectively, these findings underscore the importance of PD-1 co-inhibition in transplant immunology.

Organ transplant recipients have a higher cancer risk than the general population, and in these patients, cancer remains the second most common cause of death (126). Some organ transplant patients developing cancer are treated with ICIs. In contrast, ICIs can increase the risk of acute rejection related to T-type cellular immunity activation, and immunosuppressants can compromise the antitumor activity of immunotherapy (127). Therefore, the safety and efficacy of ICIs in organ transplant recipients should be discussed thoroughly. Data pooled from the literature demonstrated that the overall allograft rejection rate is 36%-41% in organ transplant recipients after ICI therapy; in particular, graft rejection rate is 40%-44% for kidney transplant recipients (126, 128). The highest risk is noted in patients treated with PD-1 inhibitors than in those treated with PD-L1 and CTLA-4 inhibitors, whereby nivolumab demonstrated the highest rejection rate, followed by pembrolizumab (126, 128). A multicenter retrospective study in kidney transplant recipients demonstrated that 42% of the patients receiving ICIs developed acute rejection within a median onset of <4 weeks, 65.5% required dialysis, and 27.5% lost their allograft; in contrast, the acute rejection rate was 5.4% in patients not treated with ICIs (129). Moreover, 50% of the rejections were T-cell-mediated rejection and the rest were mixed (T-cell and antibody mediated rejection) in their series of kidney transplant patients treated with ICIs. Suzuki et al. identified PD-1 agonists inhibiting T cells by triggering immunosuppressive signaling in murine disease models with acute graft versus host disease and colitis and indicated their clinical potential for treating issues related to allogeneic transplantation (130). Taken together, these results indicate the necessity of considering both rejection risks and objective response rates and maintaining two-agent immunosuppression regimens to achieve reasonable tumor response with a low rejection risk.

### 3.4 Renal aging

Senescent cell accumulation within tissues is a hallmark of the aging process. The immune system can clear senescent cells as part of normal tissue homeostasis. However, the excessive generation and insufficient elimination of senescent cells in various tissues promote inflammation and potentially contribute to pathological aging (131). Senescent cells expressing PD-L1 can escape immune surveillance, resulting in their accumulation and associated inflammation (132, 133). PD-L1 mRNA upregulation in bone, heart, liver, marrow, and lung has been observed in aged mice (132). Wang et al. recently reported an increase in PD-L1^+^ cell population among tdTomato^−^ cells from the liver and kidneys of aged mice. The author also reported that anti-PD-1 administration reduced the total number of p16^+^ cells and the population of PD-L1^+^ cells in an activated CD8^+^ T-cell-dependent manner in naturally aging mice or a mouse model with normal livers or induced nonalcoholic steatohepatitis *in vivo*, ameliorating various aging-related phenotypes (134). Therefore, PD-1 and its ligands (PD-L1 and PD-L2) are overexpressed after podocyte injury and during cellular senescence. PD-1 upregulation activates caspase-3 and induces metabolic disruption, leading to podocyte injury and loss. Anti-PD-1 ameliorates these effects *in vitro* and *in vivo* (19, 134, 135). Preventing aging via PD-1 immune checkpoint blockade may be a promising therapeutic strategy.

Regardless of the cause of podocyte disease, lost podocytes are irreplaceable because they are unique, highly specialized, terminally differentiated, and restricted in a postmitotic state with limited repair or regeneration ability (136, 137). Podocyte number and density decrease with advancing age (138, 139). Global transcriptomic changes that occur in aged mouse podocytes indicate that decreased expression of canonical podocyte marker genes, junctional and adhesion proteins, and prosurvival pathway proteins synergizes with an increase in the activities of inflammatory response pathways and a decrease in those of podocyte-specific signaling (139). Pippin et al. reported that the PD-1 pathway activity increases in aged mouse and human glomeruli and that it is correlated with glomerular scarring, vascular damage, and declined kidney function (19). Moreover, aged mouse and human kidneys displayed higher PD-1 levels in podocytes, parietal epithelial cells, and tubular cells but not in glomerular mesangial cells and endothelial cells (19). In an *in vitro* study, interfering with PD-1 signaling in mice by using neutralizing anti-PD-1 significantly improved the aging phenotype in the glomeruli, tubular epithelial cells, and the tubulointerstitium in the kidneys, as well as in the liver, and increased podocyte lifespan (19).

Age-related diseases are progressive conditions, typically involving inflammation and fibrotic degeneration. Emerging studies have indicated that the PD-1/PD-L1 axis plays a critical role in these diseases associated with the accumulation of senescent cells with inflammatory and degenerative alterations, such as those in atherosclerosis, chronic obstructive lung disease, coronary artery disease, and Alzheimer’s disease (133, 140–143). In fibrotic processes, fibroblasts are the primary source of myofibroblasts. PD-1/PD-L1 signaling mediates fibrotic pathological responses by modulating T-cell immunity, fibroblast activation, and epithelial-mesenchymal transition (34, 144). In the fibrotic tissues of both mice and humans, fibroblasts, endothelial cells, and epithelial cells demonstrate high PD-L1 expression (34, 132, 145–147). Moreover, tubulointerstitial nephritis (TIN) and renal fibrosis have been noted to occur after administration of PD-1 or PD-L1 inhibitors for treating various cancers (34, 148).

### 3.5 Renal complication secondary to PD-1 immunotherapy

The increase in clinical use of ICIs has led to an increase in the number of reported irAEs because ICIs exuberantly activate immune responses. The renal complications of ICI administration mainly include acute kidney injury (AKI), proteinuria, and electrolyte abnormalities, all attributable to renal or extrarenal irAEs. Compared with extrarenal irAEs, intrarenal irAEs are less common (149, 150). In a retrospective cohort study, 17% of patients who received PD-L1 inhibitors for 1 year developed AKI, and 6% developed sustained AKI; however, only <1% of the patients were suspected to have PD-L1-related AKI (151). In clinical trials on PD-1 inhibitors, the pooled incidence of all and grade 3-4 AKIs has been 2.2% and 19%, respectively (152). The mechanisms underlying ICI-associated AKIs include reactivation of effector T cells, loss of tolerance versus self-renal antigens, increase in PD-L1 expression by tubular cells, or establishment of a proinflammatory milieu with self-reactive antibody release (149). These AKIs are independent of the drug dose received, but they differ based on the drug type (152). The incidence of AKIs associated with anti-CTLA-4 + anti-PD-1 therapy (4.9%) is higher than that of AKIs associated with anti-PD-1 monotherapy, including nivolumab (2.3%) and pembrolizumab (2.0%) and that of AKIs associated with anti-PD-L1, including atezolizumab (1%), durvalumab (1%), or avelumab (0%) (151, 152). Available pharmacokinetic data have revealed that ICIs are cleared primarily by nonspecific proteolytic degradation in the plasma and peripheral tissues, not in the liver or kidneys (150). As such, ICI dose need not be adjusted for the prevention of kidney impairment; however, patients with high-grade nonselective proteinuria may develop impaired efficacy because of drug clearance through urinary loss (150).

Acute TIN (ATIN) is a predominant lesion observed in the kidney biopsies of 93% of ICI-related AKI cases (153). In their meta-analysis of clinical trials, Manohar et al. reported an estimated acute interstitial nephritis (AIN) rate of 16.6% among patients who developed AKI after treatment with PD-1 inhibitors; in particular, the AIN rates were 15% and 21.6% for nivolumab and pembrolizumab, respectively (152). Izzedine et al. observed that 33.3% of pembrolizumab-treated patients demonstrated AIN in renal biopsy and that pembrolizumab withdrawal coupled with corticosteroid therapy most effectively led to kidney function recovery, proteinuria improvement, or both (154). The lesions in renal tubules and interstitium present ATIN, alone or in combination with glomerulopathies (155). Hakroush et al. reported that all renal biopsies with AIN related to ICI therapy and 27.9% of ICI-naïve renal biopsies with underlying kidney diseases were positive for PD-L1, whereas all control kidneys with nephrectomy were PD-L1 negative (156). Both tubular and glomerular PD-L1 positivity occurs in patients with ICI-related AIN. However, Cassol et al. demonstrated that tubular epithelial cell membrane was positive for PD-L1 only in patients with PD-1 inhibitor-associated AIN but not in those with acute tubular necrosis or AIN secondary to other medications (157). Relatively few data, mainly in the form of case series, are available on glomerular diseases associated with ICIs (21, 22, 158–160). A systematic review indicated the most frequent biopsy-confirmed ICI-associated glomerular diseases were pauci-immune glomerulonephritis/renal vasculitis (27%), podocytopathies (24%), and complement 3 GN (11%) (161). Most patients demonstrated full or partial recovery after discontinuing ICIs or receiving corticosteroid treatment; however, 19% of the patients remained dialysis-dependent, and approximately one-third died. Other nephrotoxicities in the form of glomerular injury included IgA nephropathy, membranous nephropathy, antiglomerular basement membrane disease, lupus-like nephritis, thrombotic microangiopathy, and amyloid A amyloidosis (155, 161, 162). As such, distinguishing renal irAEs from other causes of AKI is critical, and kidney biopsy should be only considered when ICI-associated nephrotoxicity is suspected.

## 4 Conclusion

PD-1 and its ligands, representative immunosuppressive checkpoints, constitute an inhibitory pathway mediating immune tolerance and affording immune homeostasis. Recent years have enabled a rapid expansion of the current knowledge regarding PD-1 immunology, which involves cancer immunity, autoimmunity, infection immunity, transplantation immunity, allergy, immune privilege, and PD-1/PD-L1 inhibitor-related irAEs. Major advances have been made in immunotherapy for immune-associated disorders, particularly cancer therapy; moreover, therapies involving PD-1/PD-L1 blockade have been approved for the treatment of various cancers.

In this review, we highlighted the growing relationship between PD-1 immunology and the kidneys. PD-1 receptors, PD-1 ligands, or both on immune cells (kidney macrophages, DCs, and lymphocytes) and renal parenchymal cells (proximal tubule epithelial cells and podocytes) can aid in maintaining immunological homeostasis in the kidneys. Understanding these interconnected networks between PD-1 immunology and distinct cell populations related to renal cell carcinoma, glomerulonephritis, kidney transplantation, or renal aging will be critical to the development of novel drugs targeting PD-1 signaling. Despite the efficacy of PD-1/PD-L1 inhibitors in cancer therapy, several molecular targeted drugs have been implicated in the development of renal complications, which can range from distinct renal irAEs to extrarenal irAEs. Consequently, PD-1 and its ligands have significant roles in the kidneys, necessitating further mechanistic and clinical studies to delve deeper into their implications.
